# Supplementary material for: Comparative genomics provides new insights into the diversity, physiology, and sexuality of the only industrially exploited tremellomycete: Phaffia rhodozyma
Source: BMC Genomics. 2016 Nov 9;17:901. doi: 10.1186/s12864-016-3244-7 (PMC5103461; doi:10.1186/s12864-016-3244-7)
Supplement: Additional file 6: — List of orphan genes with links to PFAM (related to Additional file 1: Table S1). (ZIP 1428 kb) [file 12864_2016_3244_MOESM6_ESM.zip › PFAM_UNIQUE/G02653_P.html]

#                                                                            --- full sequence --- -------------- this domain -------------   hmm coord   ali coord   env coord
  
# target name        accession   tlen query name           accession   qlen   E-value  score  bias   #  of  c-Evalue  i-Evalue  score  bias  from    to  from    to  from    to  acc description of target
  
#------------------- ---------- ----- -------------------- ---------- ----- --------- ------ ----- --- --- --------- --------- ------ ----- ----- ----- ----- ----- ----- ----- ---- ---------------------
  
G02653\_P             -            224 CBFB\_NFYA            PF02045.10    58      0.67    9.2   7.0   1   1   0.00042       1.3    8.3   7.0     9    52   182   223   178   223 0.90 - CCAAT-binding transcription factor (CBF-B/NF-YA) subunit B  
G02653\_P             -            224 ETS\_PEA3\_N           PF04621.8    341       5.9    4.7   7.8   1   1   0.00098       1.9    6.3   5.1    93   159   137   196   120   219 0.60 - PEA3 subfamily ETS-domain transcription factor N terminal domain  
G02653\_P             -            224 IL12p40\_C            PF10420.4     82       7.1    5.5  16.5   1   1         1     6e+03   -4.7  16.5     8    38    37   100    31   204 0.64 - Cytokine interleukin-12p40 C-terminus
